# Structural and biochemical basis for the formation of α-(1 → 3)-linked mannose residue in *O*-glycans of *Saccharomyces cerevisiae*

**DOI:** 10.1016/j.jbc.2026.113626

**Published:** 2026-10-08

**Authors:** Daisuke Hira, Chihiro Kadooka, Takuji Oka

**Affiliations:** Department of Biotechnology and Life Sciences, Faculty of Biotechnology and Life Sciences, Sojo University, Kumamoto, Japan

**Keywords:** mannosyltransferase, molecular dynamics, glycosyltransferase, protein structure, *Saccharomyces cerevisiae*

## Abstract

The biosynthesis of *O*-mannose-type glycans in the budding yeast *Saccharomyces cerevisiae* requires the sequential action of multiple mannosyltransferases. However, the molecular mechanism governing α-(1 → 3)-linkage formation remains poorly understood. Mnt2p, a member of glycosyltransferase family 71 (GT71), catalyzes the transfer of the fourth and fifth mannose residues of *O*-glycans *via* α-(1 → 3)-linkages. Here, we report the crystal structure of Mnt2p at 2.40 Å resolution, representing the first experimentally determined structure of a GT71 family enzyme. The structure revealed an N-terminal domain adopting a canonical glycosyltransferase family A fold harboring a DXD motif that coordinates a Mn^2+^ ion and a novel C-terminal domain unique to the GT71 family. The active site is formed at the interface between these two domains, and GDP-mannose binds to a cleft adjacent to the catalytic metal ion. Using recombinant Mnt2p expressed in *Escherichia coli*, we biochemically demonstrated its α-(1 → 3)-mannosyltransferase activity and determined its substrate specificity toward α-mannobiose acceptors. Docking and molecular dynamics simulations revealed that the C-terminal domain forms the acceptor substrate-binding site, providing the structural basis for α-(1 → 3)-linkage selectivity, distinct from the linkage specificity of α-(1 → 2)-mannosyltransferases. These findings establish the structural foundation for understanding the differentiation of α-(1 → 2) and α-(1 → 3) mannosyl linkages in yeast *O*-glycan biosynthesis.

In addition to physically protecting the cell and defining its shape, the fungal cell wall functions to exchange molecules, sense signals, and communicate with other cells (1). The cell wall of the budding yeast *Saccharomyces cerevisiae* is constructed by complex cross-linking of β-(1 → 3)- and β-(1 → 6)-glucans, a small amount of chitin, and many proteins that may bear *N*- and *O*-linked glycans and a glycolipid anchor (1). Mannans are found in the cell walls of *S. cerevisiae*, and their biosynthesis has been studied extensively 1, 2, 3, 4.

*O*-Mannose-type glycans were first identified in *S. cerevisiae* and are frequently modified in secretory and cell wall proteins. These short oligomannose chains are attached to the hydroxyl group of a serine or threonine residue on a protein *via* a glycosidic bond in an α-anomeric arrangement (5). In the endoplasmic reticulum (ER), Dol-P-Man is synthesized by the membrane protein complex Dol-P-Man synthase at the cytosolic face of the ER membrane (6). The mannosyl transfer of the reducing terminal mannose residue occurs in the ER. *O*-Mannosylation initiates with the transfer of a mannose residue from Dol-P-Man to Ser and/or Thr residues of translated polypeptides, catalyzed by the PMT family (7). The second and subsequent mannosyl residues are extended stepwise in the Golgi apparatus by α-(1 → 2)-mannosyltransferases of the KTR family (Kre2p, Ktr1p, and Ktr3p), forming α-(1 → 2)-linked mannobiose mannotrioses 8, 9. Subsequently, the MNN1 family of α-(1 → 3)-mannosyltransferases (Mnn1p, Mnt2p, and Mnt3p) transfers additional α-(1 → 3)-mannosyl residues in the Golgi, forming linear oligosaccharides often up to five mannosyl residues in length (10). In addition, mannosyl phosphate is incorporated by the mannosyl phosphotransferases Mnn4 and/or Mnn14 11, 12.

Mnt2p is a Golgi-localized α-(1 → 3)-mannosyltransferase that transfers the fourth and fifth mannose residues of *O*-glycans in *S. cerevisiae*. Mnt2p is more strongly involved in the transfer of the fifth mannose residue, as shown by genetic analyses (10). However, enzymatic studies of Mnt2p are limited, and the enzymatic activity of purified Mnt2p has not been demonstrated biochemically *in vitro*. Mnt2p is a member of the glycosyltransferase family glycosyltransferase family 71 (GT71), and Mnn1p, Mnt3p, Mnt4p, Mnn2p, and Mnn5p are known paralogs in budding yeast (4). Mnn1p, Mnt2p, and Mnt3p have been identified as α-(1 → 3)-mannosyltransferases based on changes in glycan structures in gene-disruption strains (10). Mnn2p and Mnn5p are α-(1 → 2)-mannosyltransferases that synthesize the outer sugar chains of N-glycans in *S. cerevisiae* (13).

Structural studies of glycosyltransferases have revealed that variable loops inserted into conserved core folds serve as templates for diverse acceptor substrate specificities (14). Crystal structures have been determined for a few fungal mannosyltransferases. For example, the α-(1 → 2)-mannosyltransferase structures of Mnt1p and Ktr4p of budding yeast and CmsA/Ktr4 of *Aspergillus fumigatus*, which belong to the GT15 family, have been elucidated 15, 16, 17, 18. Furthermore, the protein structure of Mnn9, which belongs to GT62 and is part of the M-Pol-I complex involved in the biosynthesis of α-(1 → 6)-linked mannan of *N*-glycans in budding yeast, has been determined (19). However, the protein structures of enzymes belonging to the GT71 family and α-(1 → 3)-mannosyltransferases have not been determined. Although GT71 family members, including Mnn1p, Mnt2p, Mnt3p, and Mnt4p, function as α-(1 → 3)-mannosyltransferases based on glycan structure analyses of gene-disruption strains, few studies have reported enzyme activity levels using recombinant proteins (10). The KTR family enzymes (Kre2p, Ktr1p, and Ktr3p) add the second and third α-(1 → 2)-linked mannose residues, and the fourth and subsequent mannose residues are added by α-(1 → 3)-mannosyltransferases in the GT71 family. Although the localization of glycosyltransferases and lipid-raft-like membrane microdomains are thought to be factors, it is not clear why the α-(1 → 3)-mannose chain is not formed at the second or third position (*i.e.*, how the α-(1 → 2) and α-(1 → 3) linkages are differentially formed). A study showing that not only α-(1 → 2)-mannosyltransferases of the KTR family but also α-(1 → 3)-mannosyltransferases, including Mnt2p, are abundant in the early Golgi fraction does not support the theory that the expression and transport of these enzymes are regulated to create the mannose chain structure (20). Biochemical studies and structural elucidation of the GT71 family of α-(1 → 3)-mannosyltransferases could help identify the factors responsible for the differentiation of α-(1 → 2) and α-(1 → 3) bonds in *O*-glycans.

In this study, we obtained Mnt2p as a recombinant protein using an *Escherichia coli* expression system and biochemically demonstrated that Mnt2p is an α-(1 → 3)-mannosyltransferase through structural analyses of the enzymatic reaction products. We also determined the X-ray crystal structure of Mnt2p, which represents the first experimentally determined structure of a GT71 family enzyme and an α-(1 → 3)-mannosyltransferase. Furthermore, we performed molecular dynamics (MD) simulations of the enzyme–substrate complex to investigate the catalytic mechanism. This study provides a structural basis for understanding the differentiation of α-(1 → 2) and α-(1 → 3) bonds in *S. cerevisiae O*-glycans.

## Results

### Expression and purification of Mnt2p in *Escherichia. coli*

To our knowledge, an active form of recombinant Mnt2p has not been obtained using any expression system, including *E. coli* and *Pichia pastoris*. We chose the *E. coli* expression system over yeast to obtain a homogeneous, unglycosylated protein sample, as the heterogeneous glycan modifications typical of yeast expression severely hinder crystallization. Although the success rate for the soluble expression of glycosyltransferases in *E. coli* is generally low, we successfully optimized the conditions to achieve excellent yields. To obtain a recombinant Mnt2p protein using the *E. coli* expression system, the gene encoding the soluble form of Mnt2p lacking an N-terminal region (amino acids 1–25), including both a putative signal sequence and transmembrane domain, was introduced into an *E. coli* expression vector fused in-frame with a 6x histidine (6xHis) tag at the N-terminus. Protein expression was successfully achieved by culturing the cells at a low temperature. The purified Mnt2p was analyzed using SDS-PAGE (Fig. 1*A*). Mnt2p was visualized as a band of approximately 60 kDa, similar to the expected molecular weight of 63.5 kDa for Mnt2p lacking an N-terminal region.

**Figure 1:**
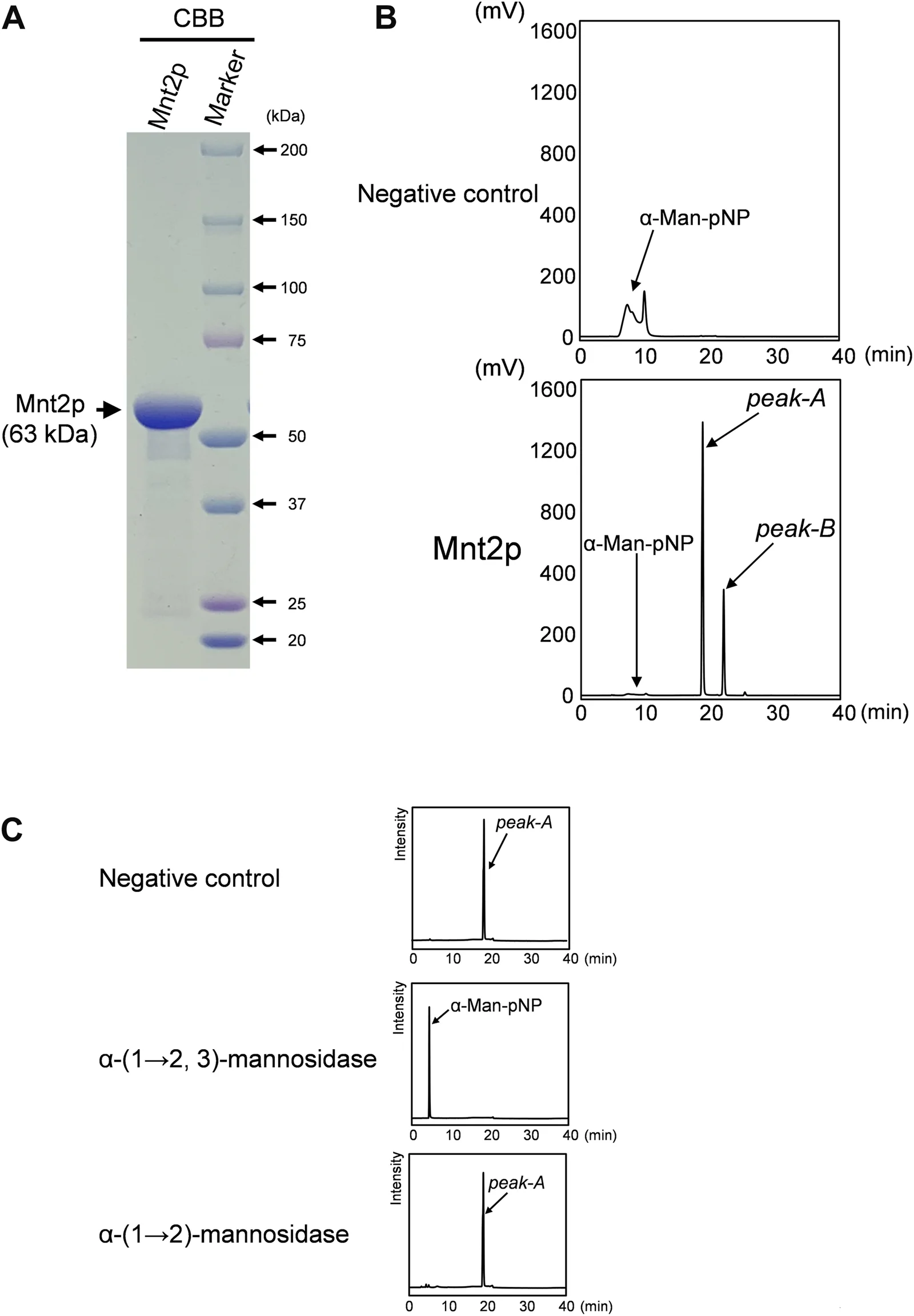
**Expression of Mnt2p and *in vitro* assay of Mnt2p mannosyltransferase activity.***A*, purified recombinant Mnt2p (5 μg) was separated by 5 to 20% SDS-PAGE and stained with Coomassie brilliant *blue,* revealing bands of approximately 60 kDa. *B*, *In vitro* assay of Mnt2p mannosyltransferase activity. Chromatograms of *in vitro* Mnt2p mannosyltransferase activity assays using *p*-nitrophenyl α-d-mannopyranoside as the substrate. A reaction mixture (40 μl) containing 25 mM Hepes-NaOH (pH 7.0), 50 mM NaCl, 15 mM KCl, 2.5% glycerol, 1 mM MnCl_2_, 1.5 mM α-Man-pNP (acceptor), 5 mM GDP-Man *(donor)*, and 4.0 μg of purified Mnt2p was incubated at 30 °C for 12 h. Chromatograms on the *left* show typical results of the assay without Mnt2p (−Mnt2p, *upper panel*) and with Mnt2p (+Mnt2p, *lower panel*). Assays without Mnt2p yielded only peaks from the substrate (α-Man-pNP), which splits into two peaks (∼8 and ∼10 min) on the amino column (17), while fractions with Mnt2p showed a decrease in these substrate peaks and contained new reaction peaks (termed peak-A and peak-B) at 18.0 and 20.4 min. *C*, determination of peak-A structure using substrate-specific mannosidases. The *upper panels* show chromatographs of purified peak *A*. Purified peak A was digested by α-(1 → 2, 3)-specific mannosidase *(middle panels)* and α-(1 → 2)-specific mannosidase *(lower panels)*. Peak-A could be digested only by α-(1 → 2, 3)-specific mannosidase and converted to α-Man-pNP. *α-Man-pNP, p*-nitrophenyl α-d-mannopyranoside.

### Recombinant Mnt2p has α-(1 → 3)-mannosyltransferase activity *in vitro*

Genetic analyses of a Δ*mnt2* strain have demonstrated that Mnt2 functions as an α-1,3-mannosyltransferase based on the altered *O*-glycan structure (10). However, to our knowledge, no study has directly shown that the enzymatic reaction product contains an α-1,3-mannose linkage. Accordingly, we evaluated whether Mnt2p has α-1,3-mannosyltransferase activity. Firstly, we measured mannosyltransferase activity at 30 °C using the chemically synthesized substrate *p*-nitrophenyl α-d-mannopyranoside (α-Man-pNP, 1.5 mM) as a sugar acceptor, guanosine diphosphate-α-d-mannose (GDP-Man, 5 mM) as a sugar donor, and 1 mM Mn^2+^ as a cofactor (Fig. 1*B*). The reaction mixture was analyzed using HPLC with an amino column, and products were detected by UV absorbance at 300 nm. Reaction mixtures incubated for 12 h without Mnt2p did not yield any additional peaks on the chromatogram, although the acceptor substrate α-Man-pNP eluted as two distinct peaks (∼8 and ∼10 min) due to a known chromatographic phenomenon on amino columns (17), which was confirmed by electrospray ionization (ESI)-MS analysis to be a single mass corresponding to α-Man-pNP (Fig. S4). The reaction mixture with Mnt2p showed a decrease in these substrate peaks and yielded additional peaks at 18.0 and 20.4 min, referred to as peak-A and peak-B, suggesting enzymatic reaction product formation (Fig. 1*B*). To determine the chemical structures of the reaction products, we collected the fractions and subjected them to high-resolution ESI-MS analysis. The spectra clearly yielded formate adduct ions corresponding exactly to the sequential addition of mannose residues for peak-A and peak-B (Fig. S4). The resulting digested product, α-Man-pNP, eluted as a single sharp peak at ∼4 min. As we previously reported (17), the peak splitting and delayed mobility observed in the unpurified mixture are caused by trace impurities present in the commercial pNP-Man substrate. Fractionation of the sample successfully removes these impurities, thereby resolving the peak splitting and restoring the single sharp peak. We also experimentally confirmed that the α-(1 → 2, 3)-specific mannosidase does not cleave the base pNP-linked mannose residue, which is consistent with the manufacturer's (New England Biolabs) specifications. Furthermore, peak-A was digested by α-(1 → 2, 3)-specific mannosidase, which removes terminal mannose residues linked *via* either α-(1 → 2) or α-(1 → 3) linkages, but not by α-(1 → 2)-specific mannosidase, which removes only α-(1 → 2)-linked terminal mannose residues (Fig. 1*C*). These direct mass measurements and specific-mannosidase analyses clearly confirm that peak-A and peak-B are α-Man-(1 → 3)-α-Man-pNP and α-Man-(1 → 3)-α-Man-(1 → 3)-α-Man-pNP, respectively.

### Substrate specificity of Mnt2p

To explore the properties of Mnt2p in mannosyl-chain elongation, we measured its activity on three acceptor substrates: α-(1 → 2)-mannobiose, α-(1 → 3)-mannobiose, and α-(1 → 6)-mannobiose. The mannosyltransferase activity of Mnt2p was highest when α-(1 → 3)-mannobiose was used as an acceptor substrate (54.5 nmol/min/mg of protein; Fig. 2). Mannosyltransferase activities of Mnt2p toward α-(1 → 2)-mannobiose and α-(1 → 6)-mannobiose were significantly lower (24.3% and 12.7%, respectively) compared to that toward α-(1 → 3)-mannobiose (*p* < 0.05, Fig. 2). This substrate specificity is consistent with that reported by Romero *et al.* (10) and supports the role of Mnt2p in transferring the fourth and fifth α-(1 → 3)-linked mannose residues of *S. cerevisiae O*-glycans.

**Figure 2:**
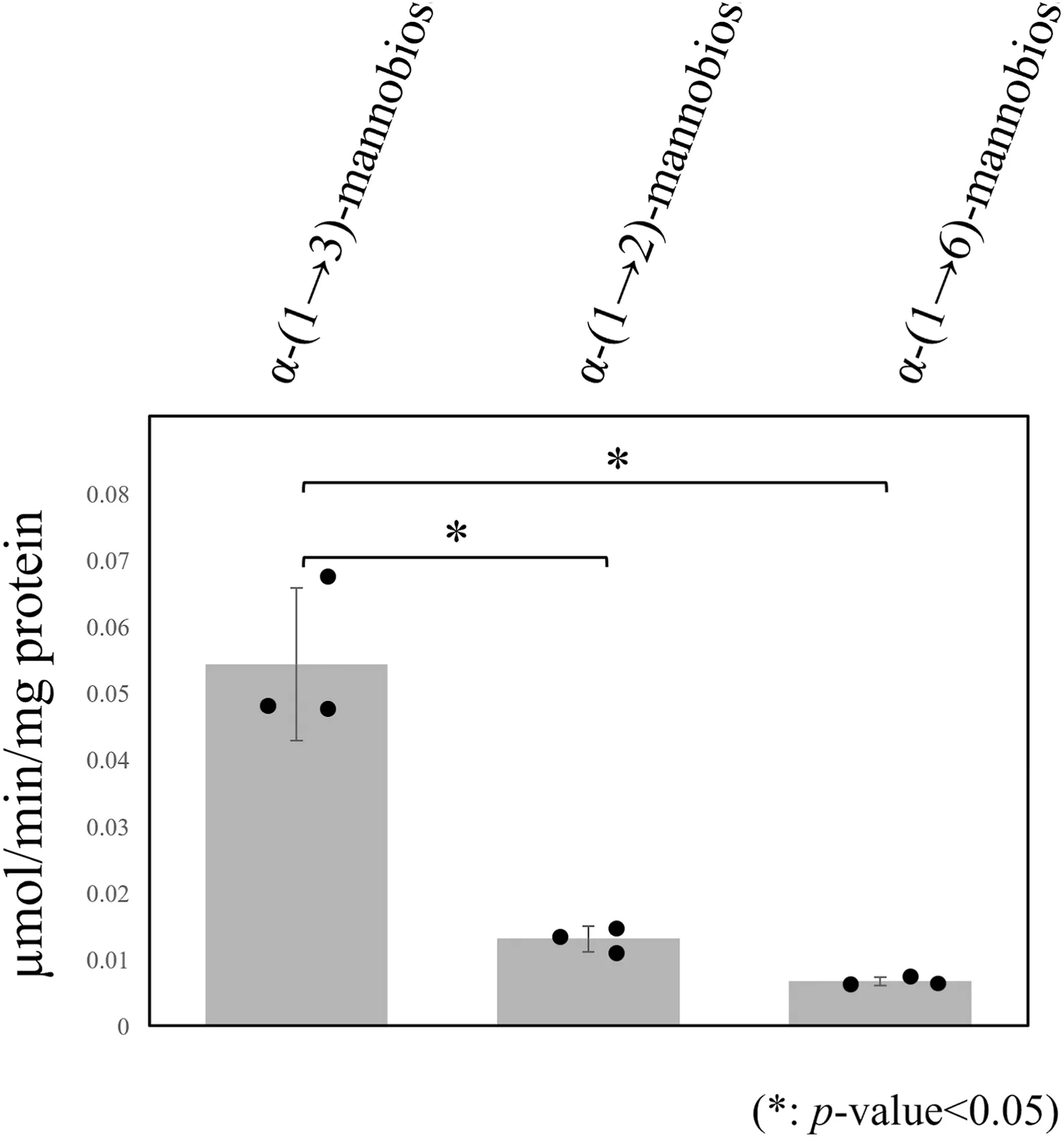
**Substrate specificity of Mnt2p.** A reaction mixture (40 μl) containing 25 mM Hepes-NaOH (pH 6.8), 50 mM NaCl, 15 mM KCl, 2.5% glycerol, 1 mM MnCl_2_, 5 mM GDP-Man (donor substrate), 1.63 μg of purified Mnt2p, and 2.5 mM α-(1 → 2)-mannobiose, α-(1 → 6)-mannobiose, or α-(1 → 3)-mannobiose (acceptor substrates) was incubated at 30 °C for 60 min. Each reaction mixture was labeled with ethyl 4-aminobenzoate and then analyzed using HPLC. Data are presented as the mean ± SD of three independent experiments, with individual data points superimposed on the bar graph. Statistical significance was analyzed using Welch's *t* test, comparing the activity toward the α-(1 → 3)-mannobiose acceptor against the α-(1 → 2)- and α-(1 → 6)-mannobiose substrates (∗: *p*-value < 0.05).

### Structural determination of Mn^2+^/GDP-Man Mnt2p complexes

Mnt2p protein crystals suitable for X-ray data collection emerged after 1 week at 4 °C from a solution containing Mn^2+^ as the metal ion and GDP-Man as the donor substrate. The crystals diffracted X-rays to 2.40 Å resolution, with space group P2_1_2_1_2_1_ and unit cell sizes a = 130.9 Å, b = 131.2 Å, and c = 169.6 Å. Molecular replacement using a polyalanine structural model generated from the AlphaFold2-predicted structure successfully placed four Mnt2p monomers, with a Matthews coefficient of 2.87 and solvent content of 57%. During structural refinement, electron densities assigned to Mn^2+^ were identified in the region in contact with the side chains of Asp-277, Asp-279, and Asn-459, and electron densities attributed to GDP-Man were also present in the vicinity. Finally, we obtained a refined structure consisting of four Mnt2p molecules, each bound to one active-center Mn^2+^ ion and one donor substrate, GDP-Man. The final *R* and *R*_free_ values were 0.206 and 0.233, respectively, and the RMSD for bond lengths and angles from ideal values were 0.006 Å and 1.33°, respectively (Table S1).

### Overall Mnt2p structure

The crystal structure of Mnt2p contained four monomers in the asymmetric unit and was well-defined in the electron density map. The proteins were continuously modeled, except for residues 89 to 94. These residues form a loop connecting the α-2 and α-3 helices. The RMSD for the Cα atom positions between protein molecule pairs in the asymmetric unit of the crystals was 0.24 to 0.46 Å after superposition of the common Cα atoms, and all monomer molecules had identical structures except for two loop regions with a slightly higher temperature factor on the molecular surface consisting of residues 60 to 67 and 429 to 437.

The Mnt2p monomer included an N-terminal domain with a mixed α/β structure common to the glycosyltransferase family A (GT-A) possessing a DXD motif and a C-terminal domain specific to Mnt2p-like proteins. These domains faced each other, forming the active site at their interface, as illustrated in Figure 3*A*. The N-terminal domain of Mnt2p was comprised of a mixed α/β-fold containing sixteen α-helices, four 3_10_-helices, and ten β-strands, as determined using DSSP (21). The core structure consisted of a β-sheet, with eight β-strands arranged in the order β-2, β-1, β-7, β-6, β-5, β-8, β-10, and β-9, with β-1 and β-10 being antiparallel. This β-sheet was surrounded by helices and one antiparallel β-sheet (β-3 and β-4). The N-terminal domain structure of Mnt2p was compared with known experimentally determined protein structures in the Protein Data Bank (PDB) using DALI (http://ekhidna2.biocenter.helsinki.fi/dali/) (22). The proteins with high Z-scores were the catalytic domain of UDP-glucose:glycoprotein glucosyltransferase (UGGT; GT24) from *Chaetomium thermophilum* (Z-score = 12.4, RMSD = 3.1 Å over 189 aligned Cα atoms, PDB code 7ZLE, chain A (23)), the bifunctional O-antigen polymerase WbbM (Z-score = 11.7, RMSD = 4.4 Å over 204 aligned Cα atoms, PDB code 6U4B, chain A (24)), in which the aligned residues lie predominantly within the C-terminal GT8 galactopyranosyltransferase domain (residues 267–502), and glycogenin-1 (GT8; Z-score = 11.1, RMSD = 2.8 Å over 161 aligned Cα atoms, PDB code 1ZCT, chain A (25)). Other diverse GT-A family proteins did not show outstanding scores. Superpositions of the three-dimensional structures of Mnt2p with these proteins are shown in Figure S1. It is evident that while these proteins partially resemble the core β-sheet and adjacent α-helices of the N-terminal domain of Mnt2p (residues 175–417), the N-terminal helices α-1 to α-5 of Mnt2p (residues 46–167) have no corresponding α-helical elements in UGGT or WbbM. Unlike Mnt2p, which uses GDP-Man as a substrate, these proteins are GT-A enzymes that use UDP-sugar as a donor substrate 23, 24, 25. Furthermore, the budding yeast α-(1 → 2)-mannosyltransferase Kre2p/Mnt1p (GT15) showed a lower score (Z-score = 9.8, RMSD = 3.7 Å over 196 aligned Cα atoms, PDB code 1S4P, chain A (15)). These results indicate that Mnt2p exhibits low structural similarity with known GT-A fold enzymes, particularly in the regions outside the core β-sheet.

**Figure 3:**
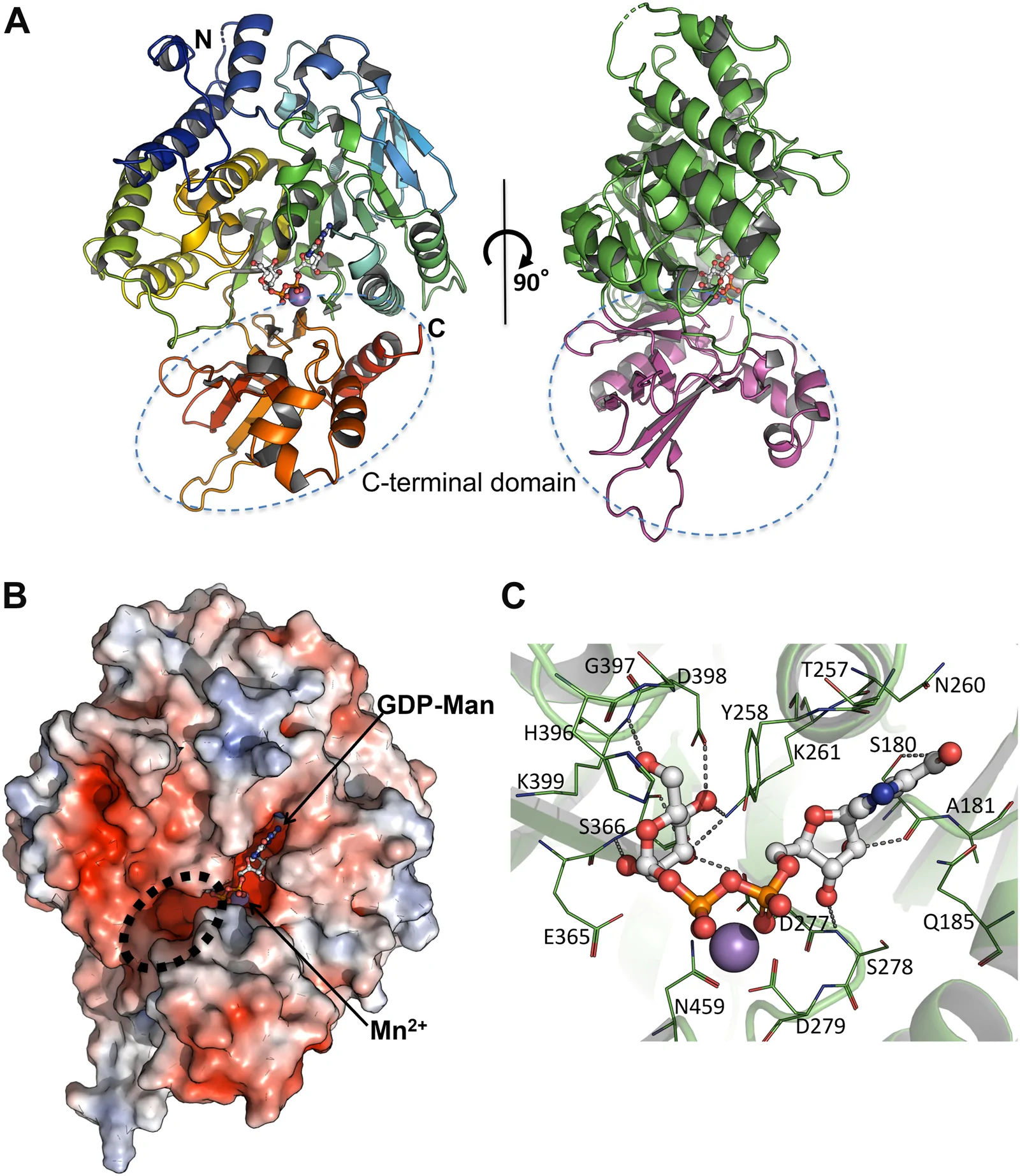
**Crystal structure of Mnt2p in complex with Mn^2+^ and GDP-Man.***A*, overall ribbon representation of Mnt2p. The N-terminal GT domain *(green)* and C-terminal domain *(magenta)* are shown. The Mn^2+^ ion is shown as a *spher*e, and GDP-Man is shown as a ball-and-stick model. The N- and C-termini are labeled. *B*, molecular surface electrostatic potential map of Mnt2p showing the cleft formed at the boundary between the N-terminal and C-terminal domains. The active site, which binds GDP-Man and Mn^2+^, is located within this cleft; *a dashed line* indicates the putative binding region for the acceptor substrate. *C*, close-up view of the active center showing the binding of Mn^2+^ and GDP-Man. The Mn^2+^ ion *(purple sphere)* is coordinated by Asp-277, Asp-279, Asn-459, and two phosphate oxygen atoms of GDP-Man. Selected residues are shown as stick models with dashed lines indicating hydrogen bonds to GDP-Man. The mannose moiety of GDP-Man is hydrogen-bonded to the main-chain amides of Ser-366 and Lys-399 and to the side chains of Lys-261, Asp-277, Ser-366, and Asp-398.

The C-terminal domain formed an α/β-fold unique to the Mnt2p protein, containing five α-helices and eight β-strands. Within this C-terminal domain fold, two disulfide bonds formed between residues Cys-442-Cys-525 and Cys-465-Cys-519. Unlike the N-terminal domain, a DALI structural similarity search using the C-terminal domain as a query did not yield any hits against experimentally determined structures in the PDB. The C-terminal domain interacts extensively with the N-terminal domain, contributing to the formation of the active center. In particular, the side chain of Asn-459 in the C-terminal domain was coordinated to the active center Mn^2+^ ion (see below).

### Mnt2p active center

As determined using electron density maps, Mn^2+^ and GDP-Man were bound to the cavity formed at the contact area between the N- and C-terminal domains (Fig. 3*B*). The Mn^2+^ ion was coordinated by Asp-277, Asp-279, Asn-459 and the two phosphate oxygens of GDP-Man (Fig. 3*C*). As with many other GT-A family proteins, Asp-277 and Asp-279 formed a DXD motif (X, any amino acid), which is essential for metal binding. Furthermore, Asn-459 in the C-terminal domain was important for divalent cation binding at the active site.

GDP-Man not only coordinated with Mn^2+^ but also interacted with protein residues. The GDP ribose group formed two hydrogen bonds, wherein its O2′ and O3′ atoms interacted with the Ser-180 and Ser-278 residues. The guanine group of GDP formed hydrogen bonds with the Ser-180 side chain and many van der Waals interactions with Ala-181, Ser-182, Gln-185, Thr-257, and Asn-260. The mannose moiety of GDP-Man was recognized by hydrogen bonds involving all four of its hydroxyl groups: the 2-OH with the main-chain amide of Ser-366, the C3-hydroxyl group (3-OH) with the side chains of Lys-261, Asp-277, and Ser-366, the 4-OH with the side chains of Lys-261 and Asp-398, and the 6-OH with the main-chain amide of Lys-399 (Fig. 3*C*).

### *In silico* analysis of the binding between Mnt2p and its acceptor substrate

To further investigate the binding of Mnt2p to the acceptor substrate, an *in silico* analysis was performed using α-Man-(1 → 3)-α-Man-OMe as a model compound for the terminal region of the mannan chain. A complex structure of the donor substrate GDP-Man and acceptor substrate α-Man-(1 → 3)-α-Man-OMe was created using AutoDock and GROMACS docking and MD simulations. Unconstrained 500 ns MD simulations were performed to introduce the substrate into the active site and examine the stability of the prepared complex. RMSDs of the main protein chain, Mn^2+^, and substrate frames and initial structures are shown in Figure S2*A*. During the first phase, the RMSD of α-Man-(1 → 3)-α-Man-OMe increased rapidly before fluctuating around 3.0 Å. This suggests that the position and structure of α-Man-(1 → 3)-α-Man-OMe deviated slightly but abruptly from the initial docked structure to form a more stable structure. Conversely, the RMSDs of the main chain atoms, Mn^2+^ ions, and GDP-Man were all less than 2.0 Å.

The last 450 ns of the MD simulation, after the system had reached equilibrium, was analyzed further. In particular, root mean square fluctuation of the main chain atoms was calculated to examine the flexibility of each residue of the polypeptide chain. The root mean square fluctuation was less than 1.6 Å except in the terminal region, indicating that the helix and β-sheet are stable, as expected. The peaks in the plot also indicated that the loop region was slightly flexible (Fig. S2*B*). These results suggest that the system is well equilibrated and the modeled complex is sufficiently stable. Fluctuations in the distances between protein residues and α-Man-(1 → 3)-α-Man-OMe in the complex were then analyzed for the last 450 ns of the MD simulation to predict hydrogen bonding. The donor–acceptor distances between the side chains of Asp-305, Arg-306, Lys-399, and Tyr-524 and the oxygen atoms of the terminal mannose residue of α-Man-(1 → 3)-α-Man-OMe remained mostly below 3.5 Å (Fig. S3). To quantify these interactions, the occupancy of each hydrogen bond, *i.e.*, the fraction of frames in which the donor–acceptor distance was ≤ 3.5 Å and the hydrogen–donor–acceptor angle was ≤ 30°, was calculated (Table S2). The hydrogen bonds of Asp-305 with the O4 and O6 hydroxyl groups of the terminal mannose residue and of Arg-306 with O6 were present in more than 95% of the frames, whereas the other hydrogen bonds, including those of Lys-399 with O3 or O4 and of Tyr-524 with the ring oxygen O5, were less persistent (37–68%). Next, RMSD-based clustering of the main-chain atoms was performed to obtain representative conformations and populations from the MD simulations (Fig. 4*A*). Figure 4*B* shows the representative conformations of the most populated cluster, where α-Man-(1 → 3)-α-Man-OMe binds to a broad cleft near the GDP-Man recognition site *via* the hydrogen bonds with Asp-305, Arg-306, Lys-399, and Tyr-524 described above (Fig. 4*C* and Table S2) and the Mnt2p C-terminal region participates in van der Waals interactions. The RMSD of α-Man-(1 → 3)-α-Man-OMe relative to its conformation in the representative structure of the most populated cluster (Fig. 4*B*) was 1.3 ± 0.5 Å over the last 450 ns, comparable to that of GDP-Man calculated in the same way (0.9 ± 0.2 Å), indicating that the acceptor occupied a stable binding pose (Fig. S2*C*).

**Figure 4:**
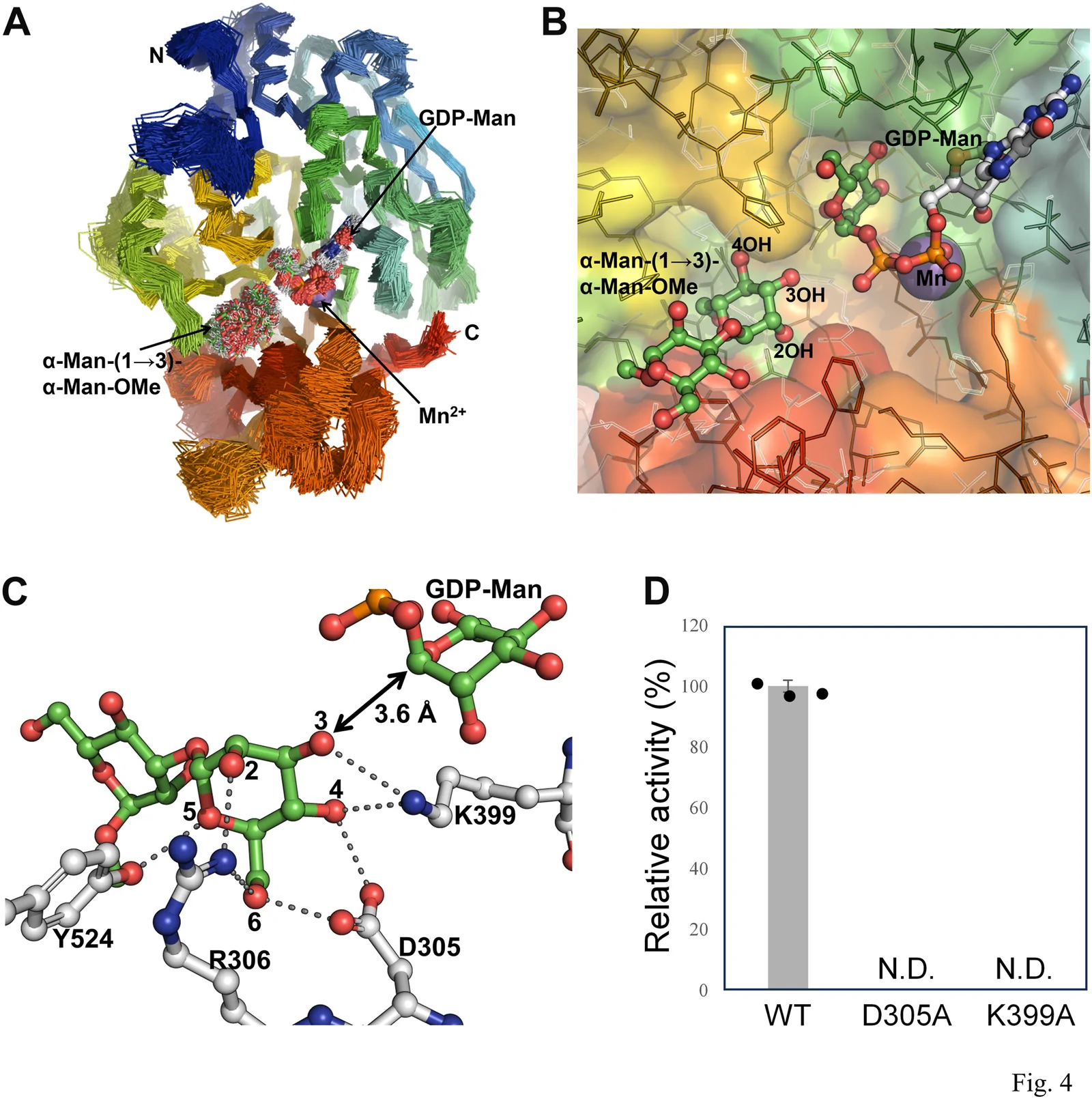
**MD simulation and mutational analysis of the acceptor-binding site of Mnt2p.***A,* superposition of representative structures from the 257 clusters. The clustering analysis based on the RMSD of the main protein chain atoms was performed on the final 450 ns of the MD trajectory, using a 1.0 Å RMSD cut-off. Mn atoms are shown as spheres. The GDP-Man and α-Man-(1 → 3)-α-Man-OMe molecules are shown as stick models. *B*, enlarged view of the molecular surface of the substrate-binding site. The substrate is recognized within the cleft formed by the N-terminal and C-terminal domains. *C*, hydrogen bonds between Asp-305, Arg-306, Lys-399, and Tyr-524 of Mnt2p and the acceptor substrate model. The *C3-hydroxyl group* group of the terminal mannose residue of the acceptor substrate is located 3.6 Å from the C1 atom of the mannose moiety of GDP-Man. *D,* mannosyltransferase activities of alanine-substitution mutants of the acceptor-binding residues shown in *C*. Activities of WT Mnt2p and the D305A and K399A mutants toward α-Man-pNP were measured by the HPLC assay under the standard assay conditions and are shown relative to that of the WT (100%). The R306A and Y524A mutants were expressed as insoluble aggregates and could not be assayed. Data are presented as the mean ± SD of three independent experiments, with individual data points superimposed on the bar graph. *α-Man-pNP, p*-nitrophenyl α-d-mannopyranoside.

In this dominant conformation, the reactive 3-OH of the terminal α-Man residue of α-Man-(1 → 3)-α-Man-OMe was located closest to the GDP-Man reaction site. According to MD simulations, the average distance between the 3-OH oxygen of the terminal α-Man and the mannose C1 atom of GDP-Man was 3.9 ± 0.4 Å over the last 450 ns (Fig. S5*A*; 3.6 Å in the representative structure), indicating a direct interaction between the donor and acceptor substrates bound to Mnt2p (Fig. 4*C*). The 3-OH approached the C1 atom from the same face as the departing GDP. The distances from the 3-OH oxygen to the bridging oxygen atom between the β-phosphate and the mannose C1 atom and to the nonbridging oxygen atom of the β-phosphate that is not coordinated to Mn^2+^ were 4.9 ± 0.4 Å and 4.6 ± 0.6 Å, respectively, and were within hydrogen-bonding distance (≤3.5 Å) in 0% and 0.3% of the frames, respectively (Fig. S5, *B* and *C*); thus, no configuration supporting a persistent hydrogen bond between the 3-OH and the β-phosphate was observed in the simulation.

### Experimental validation of the acceptor-binding site by site-directed mutagenesis

To validate the acceptor-binding model derived from MD simulations, we constructed alanine-substitution mutants of the predicted binding residues: Asp-305, Arg-306, Lys-399, and Tyr-524. We assessed the enzymatic activity of the purified soluble mutants using the established HPLC assay. The D305A and K399A mutants were successfully expressed and purified as soluble proteins, whereas the R306A and Y524A mutants were expressed as insoluble aggregates, suggesting that these residues are critical for the correct folding and structural stability of Mnt2p. As shown in Figure 4*D*, both the D305A and K399A mutants exhibited a complete loss of mannosyltransferase activity (N.D., not detectable), directly supporting the MD simulation results that these residues are essential for the acceptor-binding and catalytic function of Mnt2p.

## Discussion

In this study, we obtained the first experimentally determined three-dimensional structure of a GT71 family glycosyltransferase, Mnt2p. Using recombinant Mnt2p, we biochemically demonstrated its α-(1 → 3)-mannosyltransferase activity and identified α-(1 → 3)-mannobiose as its preferred acceptor substrate. The crystal structure, combined with molecular docking and MD simulations, established that the GT71-specific C-terminal domain plays a central role in acceptor substrate recognition, providing a structural basis for understanding how α-(1 → 3)-linkages are selectively formed during *O*-glycan biosynthesis.

The overall architecture of Mnt2p shares fundamental features with those of other characterized fungal mannosyltransferases, while exhibiting distinct characteristics that reflect the diversity of glycosyltransferases. The N-terminal domain adopted a GT-A fold, as observed in the α-(1 → 2)-mannosyltransferase Kre2p/Mnt1p (GT15) (15), and contained a DXD motif (Asp-277-Ser-278-Asp-279) that coordinates the essential Mn^2+^ ion. This conserved GT-A core is consistent with the modular evolution model in which the hydrophobic core and phosphate-binding cassette provide a structural scaffold for functional diversification (26). Consistent with this model, the N-terminal domain of Mnt2p shares its fold with the catalytic GT24 domain of UGGT, the top-scoring DALI hit, which utilizes UDP-Glc as the donor substrate (23); however, the residues that recognize the guanine base of GDP-Man in Mnt2p (Fig. 3*C*) are not conserved in UGGT. Thus, the nucleotide-sugar specificity of these structurally related but chemically distinct enzymes appears to be conferred by family-specific residues built on the common GT-A scaffold. However, the C-terminal domain of Mnt2p was structurally distinct from the α/β subdomain observed in Mnt1p. In the Mnt1p structure, the acceptor substrate-binding site is located on the protein surface and is relatively solvent-exposed (15). In Mnt2p, the C-terminal domain created a deep cleft adjacent to the donor substrate-binding site, positioning the acceptor substrate in proximity to the reactive C1 atom of GDP-Man. This architectural difference likely underlies the distinct linkage specificities of these two enzyme families: whereas Mnt1p catalyzes α-(1 → 2)-linkage formation, Mnt2p specifically catalyzes α-(1 → 3)-linkage formation.

The observation that the C-terminal domain of Mnt2p lacked structural homologs in the PDB further supports the notion that this domain evolved specifically within the GT71 family to confer α-(1 → 3)-selectivity and positional selectivity within the *O*-glycan chain. Consistent with this notion, superposition of the AlphaFold models of the other *S. cerevisiae* GT71 members on Mnt2p showed that the predicted structures of the α-(1 → 3)-mannosyltransferases Mnn1p and Mnt3p closely resemble Mnt2p, including the C-terminal domain, whereas those of the α-(1 → 2)-mannosyltransferases Mnn2p and Mnn5p superimpose poorly on Mnt2p, including the region corresponding to the C-terminal domain (Fig. S6), although these predicted structures remain to be verified experimentally. *In vitro*, Mnt2p can utilize free α-mannobiose as an acceptor substrate, demonstrating that the enzyme does not intrinsically require long glycan chains for catalysis (Fig. 2). However, *in vivo*, the acceptor substrates are not free oligosaccharides but *O*-glycan chains covalently attached to the polypeptide backbone of glycoproteins (Fig. 5). This distinction is central to understanding why α-(1 → 3)-mannosyl residues are added only at the fourth and fifth positions despite the co-localization of Mnt2p and the KTR family enzymes in the early Golgi compartments (20). The deep cleft formed by the C-terminal domain of Mnt2p (where the acceptor-substrate binding site is located) would impose structural constraints. In glycoprotein substrates, the polypeptide backbone would likely interfere with the peripheral region of the Mnt2p active site, preventing short sugar chains—such as mannose at positions 2 and 3—from reaching the catalytic center. Conversely, when the *O*-glycan chain has been extended to three or more residues by the KTR family α-(1 → 2)-mannosyltransferases, the terminal mannose would be positioned far enough from the polypeptide backbone to traverse the deep cleft and reach the donor substrate. By contrast, the surface-exposed active site of Kre2p/Mnt1p (15) would impose no such length requirement, enabling it to act on glycoprotein-bound glycans at earlier stages of chain elongation. Thus, the depth of the active-site cleft may function as a chain-length gate, ensuring the sequential order of α-(1 → 2) and α-(1 → 3) mannosylation *in vivo* (Fig. 5). This selectivity mechanism could be independent of and complementary to any spatial or temporal regulation within the Golgi apparatus.

**Figure 5:**
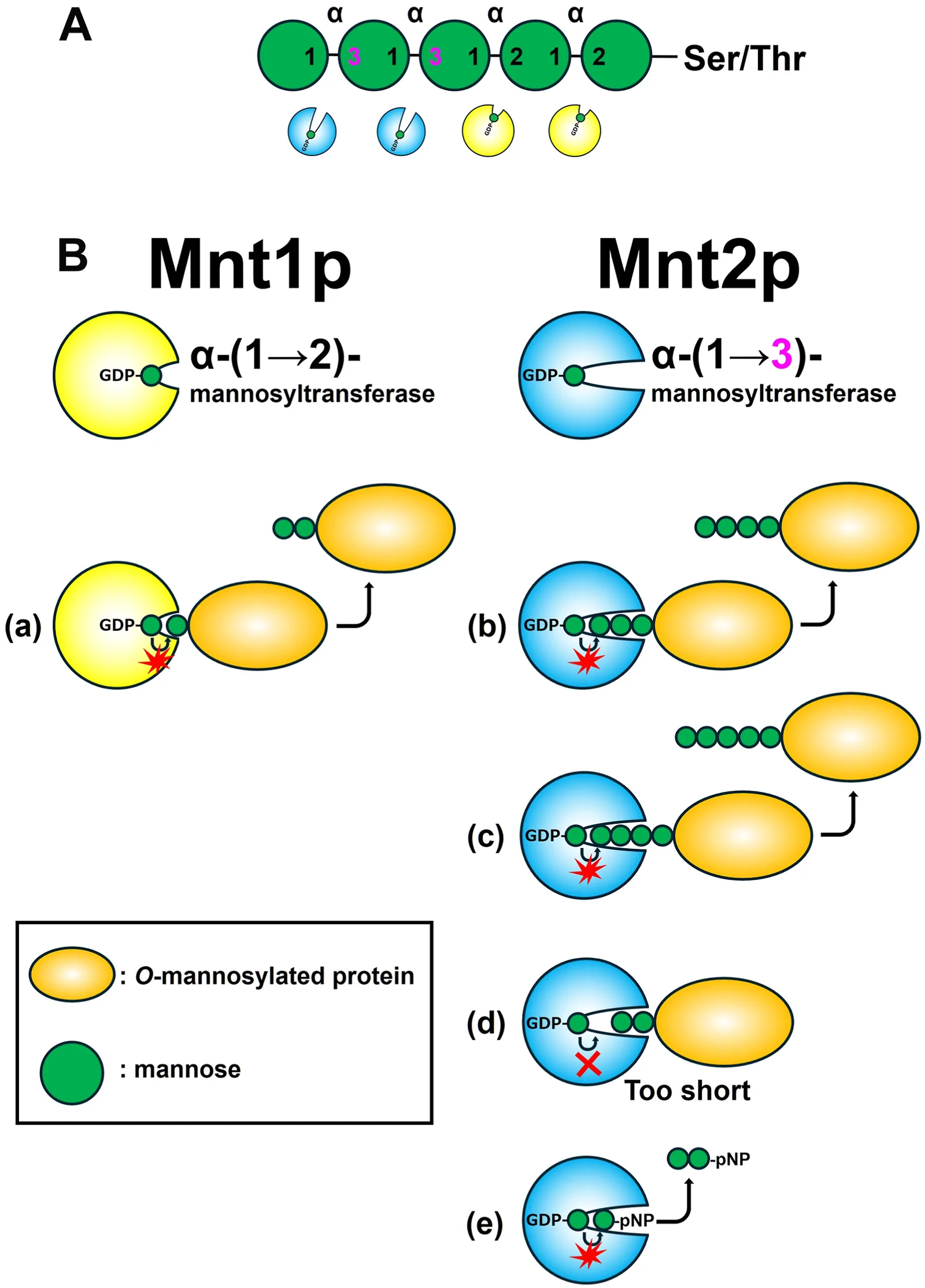
**Structure of the O-glycan chain and schematic model for the chain-length-dependent substrate specificity of Mnt1p and Mnt2p.***A*, schematic representation of a typical *O*-glycan structure in *S*. *cerevisiae*. Five mannose residues are linked to a Ser/Thr residue of the polypeptide. Numbers inside the circles indicate the linkage position (1,2 for α-(1 → 2) and 1,3 for α-(1 → 3)), and the letter α above each linkage denotes the anomeric configuration. The first and second mannose residues are added by the KTR family α-(1 → 2)-mannosyltransferases (*yellow*, including Mnt1p/Kre2p), whereas the fourth and fifth residues are added by GT71 family α-(1 → 3)-mannosyltransferases (*blue*, including Mnt2p). *B*, proposed model for the chain-length-dependent substrate selectivity. Mnt1p has an active site that is relatively exposed on its surface, whereas Mnt2p possesses a deep active-site cleft formed by its C-terminal domain. GDP-Man serves as the sugar donor for both enzymes. *Red asterisks* indicate successful mannose transfer, while *red* crosses indicate that no reaction occurred. *a,* Mnt1p transfers a mannose residue to a glycoprotein bearing a short *O*-mannosyl chain. *b* and *c*, Mnt2p transfers a mannose residue to glycoproteins bearing longer *O*-glycan chains (3 and four mannose residues, respectively). *d,* because the target polypeptide interferes sterically, the terminal mannose residue cannot reach the active site located at the *cleft bottom*; therefore, Mnt2p cannot act on glycoproteins with short *O*-glycan chains. *e,* in contrast, Mnt2p can transfer a mannose residue to free α-Man-pNP *in vitro* because this small synthetic substrate lacks bulky polypeptide moieties and can freely access the cleft. The binding modes depicted in *B* are based on the docking and MD simulations with α-Man-(1 → 3)-α-Man-OMe and have not been experimentally determined using glycoprotein substrates. *α-Man-pNP, p*-nitrophenyl α-d-mannopyranoside.

An intriguing mechanistic aspect of Mnt2p, which is intimately linked to its substrate recognition, is whether it extends the mannan chain in a processive or distributive manner. In our *in vitro* assays using the synthetic substrate, we observed a substantial accumulation of the intermediate product with a single added mannose (peak-A) alongside the completely extended product with two added mannoses (peak-B) (Fig. 1*B*). If Mnt2p were strictly processive—adding consecutive mannose residues in a single binding event without releasing the substrate—the intermediate would not be expected to accumulate to such a significant extent. This observation strongly suggests that Mnt2p operates *via* a distributive mechanism *in vitro*, releasing the glycan intermediate after each transfer reaction and requiring a rebinding event for the subsequent addition. However, as discussed above, the physiological substrates *in vivo* are growing *O*-glycan chains covalently attached to a polypeptide backbone. It remains possible that such physiological conditions and macromolecular substrates could impose local constraints that facilitate a more processive behavior. Further investigations will be required to fully elucidate the dynamic mechanism of *O*-glycan chain extension by Mnt2p *in vivo*.

The structure maintained during the MD simulation was reasonable as an enzyme-substrate complex and based on these results, we propose a reaction mechanism for Mnt2p, the first example of an α-(1 → 3)-mannosyltransferase (Fig. 6). GTs are classified as "inverting" or "retaining" depending on whether the anomeric arrangement of the C1 carbon in the monosaccharide portion transferred from the donor to the acceptor is inverted or retained. Although the general reaction mechanism of retaining GTs remains controversial and is thought to differ among families 27, 28, the docking features and MD structure of Mnt2p were consistent with the proposed catalytic mechanism of some retaining GTs. In the modeled enzyme–substrate complex, the reactive 3-OH group of the terminal mannose residue of the acceptor was positioned 3.9 ± 0.4 Å from the C1 atom of GDP-Man, and critically, approached from the same face as the departing GDP moiety. This geometry is consistent with a front-face SNi-like retaining mechanism, in which the departure of the leaving group (GDP) and the nucleophilic attack by the acceptor hydroxyl occur on the same side of the anomeric carbon, proceeding through an oxocarbenium ion-like transition state 27, 28. In the Mnt1p structure, Tyr-220 was identified as a potential nucleophilic residue that could support a double-displacement mechanism (15). However, for Mnt2p, the front-face arrangement of donor and acceptor substrates is more compatible with an SNi-like pathway, as suggested for other retaining GT-A fold enzymes 28, 29, 30. It should be noted, however, that these conclusions are based on computational modeling and are subject to inherent limitations. In particular, the acceptor-binding mode and the chain-length-dependent selectivity proposed here (Figs. 4 and 5) are derived from docking and MD simulations in the absence of an experimentally determined acceptor-bound structure, and although the loss of activity of the D305A and K399A mutants (Fig. 4*D*) supports the importance of the predicted residues, it does not define the binding mode. Moreover, the *in vitro* assays employed α-Man-pNP and free mannobioses lacking a polypeptide backbone; whether Mnt2p discriminates the chain length of O-glycans attached to glycoproteins remains to be verified. Further kinetic analyses of the mutant enzymes will be necessary to confirm the proposed recognition mechanism.

**Figure 6:**
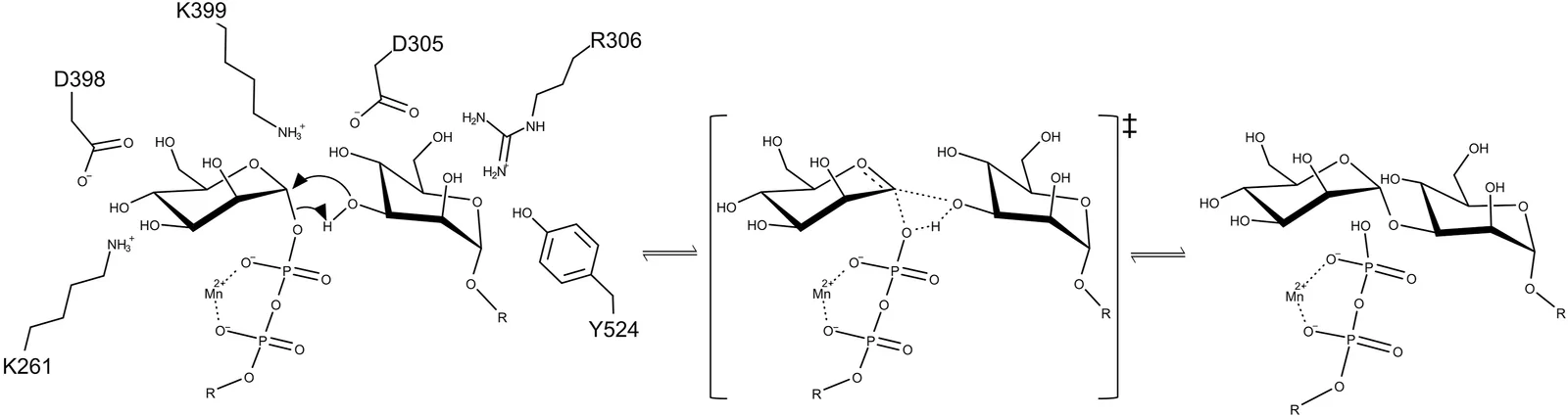
**Schematic diagram of the reaction mechanism for α-(1** **→** **3) mannosylation catalyzed by Mnt2p.***Dashed lines* indicate interatomic interactions. *Arrow*s represent electron transfer.

The structural information obtained in this study has implications for both antifungal drug development and glycoengineering. GT71 family enzymes catalyze the formation of α-(1 → 3)-mannosyl linkages that are absent in mammals, making them attractive targets for selective antifungal agents 31, 32. More broadly, mannosyltransferases involved in *O*-glycosylation are critical for fungal pathogenesis; disruption of the *Candida albicans MNT1* and *MNT2* genes, which encode α-(1 → 2)-mannosyltransferases in the KRE2/MNT1 family, results in truncated *O*-mannan, reduced adhesion, and attenuated virulence (33). Recent structural characterization of recombinant *C*. *albicans* Mnt1 has identified active-site residues amenable to inhibitor design (34). The deep cleft formed by the C-terminal domain of Mnt2p could serve as a binding site for small-molecule inhibitors with minimal off-target effects on human glycosyltransferases. Furthermore, understanding the structural basis of Mnt2p activity may facilitate the rational engineering of yeast strains with modified glycosylation patterns. The elimination of excessive mannose addition (hypermannosylation) is a key objective in the production of therapeutic glycoproteins in yeast expression systems 35, 36, and precise manipulation of GT71 family enzymes based on structural information could enable more refined control over glycan chain extension.

Several aspects of GT71-mediated mannosylation remain to be elucidated. The structures of the Mnt2p paralogs Mnn1p, Mnt3p, and Mnt4p have not yet been determined. Structural comparison of these paralogs would reveal why Mnn1p acts on both *N*- and *O*-glycans, whereas Mnt2p is specific to *O*-glycans (10). Additionally, complex structures with acceptor-substrate analogs or transition-state mimics would provide direct evidence for the proposed SNi-like mechanism and define the precise geometry of the catalytic transition state. Taken together, our findings establish the structural foundation for the GT71 family and provide a molecular framework for understanding the differentiation of α-(1 → 2) and α-(1 → 3) mannosyl linkages during fungal glycan biosynthesis. This study highlights the power of modern structural and computational biology to re-evaluate and revitalize classical pathways in fungal glycobiology, bridging historical biochemical insights with high-resolution molecular mechanisms—an approach we term "nostalgic biology".

## Experimental procedures

### Microorganisms and growth conditions

*S. cerevisiae* W303-1A (MATa *ade2-1 his3-11 leu2-3,-112 trp1-1 ura3-1 can1-100*) was used in this work. Yeast cells were cultured at 30 °C in YPAD medium containing 1% Bacto yeast extract (Difco), 2% Bacto peptone (Difco), and 0.01% adenine hemisulfate. Plasmids were amplified in *E. coli* DH5α. *E. coli* strain Rosetta-gami B (DE3) purchased from Merck Millipore was used for protein expression. Bacterial cells were cultured at 37 °C or 16 °C in Lysogeny broth (LB) containing 0.5% Bacto yeast extract (Difco), 1% Bacto tryptone (Difco), and 0.5% sodium chloride.

### Construction of Mnt2 expression vector

The expression plasmid for *MNT2* was constructed as follows. Genomic DNA was extracted from the cells of *S. cerevisiae* strain W303-1A and grown in YPDA medium for 24 h using Dr GenTLE (from Yeast) High Recovery (Takara) according to the supplier's manual. The *MNT2* gene was amplified by PCR using single-strand DNA as a template and the primers pET15b-ScMNT2-IF-F [5′-ACAGCAGCGGCCATAGCAATTCGTTAGATTTGGTAGAACCA-3′] and pET15b-ScMNT2-IF-R [5′-CACTCGAGTGCGGCCTCAAATATCTTTATTCCATATGTCGAC-3′]. The *MNT2* fragments were cloned into the NdeI and NotI sites of pET15b-KAI using the In-Fusion HD Cloning Kit (Takara) to yield pET15b-MNT2, which was transformed into Rosetta-gami B (DE3) cells (Merck Millipore). DNA sequences were confirmed by a sequence analysis using an ABI PRISM 3100 Genetic Analyzer (Applied Biosystems, Foster).

### Mnt2p expression in *E. coli* and recombinant protein purification

The gene encoding the soluble catalytic domain of Mnt2p (residues 26–C-terminus) was cloned into the pET15b-KAI vector with an N-terminal 6xHis tag and transformed into *E. coli* Rosetta-gami B (DE3) cells. Protein expression was induced with 1 mM IPTG at 16 °C for 65 h. The cells were lysed using BugBuster Protein Extraction Reagent (EMD Millipore), and the supernatant was incubated with His60 Ni Superflow Resin (TAKARA Bio) for 1 h. After washing the resin five times with buffer A (50 mM Hepes-NaOH (pH 7.0), 30 mM KCl, 100 mM NaCl, 1 mM MnCl_2_, 5% (w/v) glycerol), the protein was eluted with buffer A containing 300 mM imidazole. The eluate was dialyzed against buffer A, and the N-terminal 6xHis tag was not cleaved prior to subsequent experiments; the tag and residues 26 to 43 of Mnt2p were not visible in the electron density map of the crystal structure. The recombinant Mnt2 protein was further purified using a Hiload 16/600 Superdex 75 pg gel filtration column (GE Healthcare) equilibrated with Buffer A without glycerol (50 mM Hepes-NaOH (pH 7.0), 30 mM KCl, 100 mM NaCl, 0.1 mM MnCl_2_). Fractions containing Mnt2 were concentrated to ∼10 mg/ml in Buffer A without glycerol using a Centriprep-YM10 (Millipore) and stored in a deep freezer. Protein concentrations were determined using the Qubit Protein Assay Kit (Thermo Fisher Scientific), and purity was assessed by SDS-PAGE.

### Site-directed mutagenesis

Site-directed mutagenesis was performed using the QuikChange II Site-Directed Mutagenesis Kit (Agilent Technologies) according to the manufacturer’s instructions, using the pET15b-MNT2 plasmid as the template. The following primers were used to generate the alanine-substitution mutants: D305A [F: 5′-GCGCGGGTAATATCAGATGATC-3′; R: 5′-TGATATTACCCGCGCTTTAAAGAATAGAACTCCAG-3'], R306A [F: 5′-GCGGTAATATCAGATGATCTATTTG-3′; R: 5′-ATCTGATATTACCGCATCTTTAAAGAATAGAACTCC-3′], K399A [F: 5′-GCGGACTTTTTTTGGCTGGGCG-3′; R: 5′-CCAAAAAAAGTCCGCATCACCATGAAAATATTCTG-3'], and Y524A [F: 5′-GCGTGCACATTATTCAAGGAAGG-3′; R: 5′-GAATAATGTGCACGCATTGAACAAAAAACACTCTC-3′]. All mutations were verified by DNA sequencing. The mutant proteins were expressed and purified following the same protocol as that described for the WT Mnt2p.

### Enzyme assays

The artificial acceptor substrate α-Man-pNP was purchased from the Tokyo Chemical Industry Co., Ltd. Standard assays were performed with α-Man-pNP (1.5 mM) as the acceptor, GDP-Man (5 mM) as the donor, and purified Mnt2p (0.1 μg/μl) in a total reaction volume of 40 μl. The mixture was incubated at 30 °C for 12 h, and the reaction was stopped by heating at 99 °C for 5 min. *Para*-nitrophenol derivatives were detected by UV300 absorbance and analyzed by HPLC using a Shodex Asahipak NH2P-50 4E amino column (250 × 4.6 mm; Showa Denko) as previously described (37). α-(1 → 2, 3)-Mannosidase and α-(1 → 2)-mannosidase were purchased from New England Biolabs and ProZyme (Hayward), respectively, and used according to the manufacturers' instructions. To determine substrate specificity, α-mannobioses were used as substrate acceptors. α-(1 → 2)-Mannobiose, α-(1 → 3)-mannobiose, and α-(1 → 6)-mannobiose were purchased from Dextra Laboratories Ltd (Reading). When mannobioses were used as acceptor substrates, the reaction products were analyzed by HPLC with a fluorescence detector after labeling with ethyl 4-aminobenzoate. α-(1 → 2)-mannobiose or α-(1 → 6)-mannobiose derivatives were also labeled by the ethyl 4-aminobenzoate reaction, as described previously (37).

### High-resolution ESI-MS analysis

Molecular mass was determined using an Orbitrap Exploris 120 mass spectrometer (Thermo Fisher Scientific). The instrument was operated in negative-ion mode with full MS scans over a mass range of *m/z* 100 to 1000. Samples were dissolved in 50% (v/v) methanol containing 0.1% formic acid and directly injected into the instrument at a flow rate of 5.0 μl/min. The ESI source parameters were set as follows: spray voltage, 3.0 kV; and ion transfer tube temperature, 275 °C. Data acquisition and analysis were performed using the Xcalibur software (Thermo Fisher Scientific).

### Crystallization, data collection, and structural determination of Mnt2

Crystallization conditions were screened using the hanging-drop vapor diffusion method with Structure Screen and ProPlex Kits (Molecular Dimensions). Each droplet containing 0.8 μl of Mnt2 solution and 0.8 μl of screening solution was equilibrated against 300 μl of reservoir solution. After several rounds of optimization, Mnt2 was crystallized in a droplet containing 100 mM MOPS (pH 7.0), 8% (w/v) PEG 20000, 1.0 mM MnCl_2_, and 5.0 mM GDP-Man. The crystals were equilibrated in a solution containing 100 mM MOPS (pH 7.0), 3% PEG 20000, 1.0 mM MnCl_2_, 5.0 mM GDP-Man, and 40% glycerol (v/v) for about 3 h, frozen, and stored in liquid nitrogen. X-ray data collection was carried out at 100 K and a wavelength of 0.9000 Å using beamline BL44XU at SPring-8 (Harima). Diffraction data were integrated and scaled using XDS (38).

The crystal structure was determined by molecular replacement using MOLREP (39) from the CCP4 suite (40). The three-dimensional structure of the catalytic domain of Mnt2p (residues 26 to the C-terminus, excluding the N-terminal region containing the transmembrane helix) was predicted using AlphaFold2 (41). Of the five predicted models, the one with the highest predicted LDDT score was converted into a polyalanine model using CCP4i, and the entire polyalanine model was used as the search model. Structural refinement and several rounds of manual model building were performed using REFMAC5 (42) and Coot (43). Local noncrystallographic symmetry restraints among the four molecules in the asymmetric unit were applied in REFMAC5 throughout the refinement. The electron density of Mn^2+^ ion and GDP-Man was clearly observed, and their atomic models were built and refined using Coot and REFMAC5. The quality of the final models of the Mn^2+^/GDP-Man Mnt2 complex was assessed using PROCHECK (44) and MolProbity (45). The atomic coordinates and structural factors were deposited in the Protein Data Bank (http://www.rcsb.org/pdb) under the ID code 45PL. The data collection and refinement statistics are summarized in Table S1. Figures were generated using PyMOL (46).

### Docking simulation of the Mn^2+^/GDP-Man Mnt2 complex with the acceptor substrate

The docking simulation with methyl-α-1,3-mannobiose (α-Man-(1 → 3)-α-Man-OMe) as the acceptor substrate for the Mn^2+^/GDP-Man Mnt2 complex was performed using AutoDock 4.2 (47). The ideal α-Man-(1 → 3)-α-Man-OMe structure was built using the GLYCAM-Web server (48). The search space was defined as a cubic grid of 50 points along each axis, with 0.375 Å grid spacing, centered near the side chain of His-396 and encompassing the entire active site. Searches were performed using the Lamarckian Genetic Algorithm, with 500 runs, a population size of 150, and 2,500,000 energy evaluations. A total of 27,000 generations were analyzed and clustered using an RMS tolerance of 2.0 Å per cluster.

### MD simulation of the enzyme–substrate complex

To evaluate the dynamic properties of the modeled structure of the Mnt2 enzyme–substrate complex, we carried out MD simulations with the Mnt2 complex structure of α-Man-(1 → 3)-α-Man-OMe, one Mn^2+^ ion, one GDP-Man, and the model with the highest AutoDock docking score as the initial model. All simulations were performed using GROMACS 2019.4 (49) and the Amber ff14SB force field (50), using the previously reported topology and parameters for GDP-Man (51). The topology and parameter files for α-Man-(1 → 3)-α-Man-OMe were generated using AmberTools17's LEaP (52) with the GLYCAM06-j force field (48). The system was explicitly solvated in a cubic box using TIP3P water models, with eight Na^+^ ions added to neutralize the system's charge. Amber topologies were generated using AmberTools 17's LEaP and exported to GROMACS format with ACPYPE (53) before energy minimization by steepest descent. Following minimization, position-restrained MD simulations were performed, and the system was equilibrated in the NVT ensemble for 200 ps at 300 K using V-rescale temperature coupling, followed by 400 ps in the NPT ensemble at 1 atm using Berendsen pressure coupling. A 1.0 nm cut-off was applied for the short-range neighbor list, electrostatic, and van der Waals interactions. Long-range electrostatic interactions were computed using the Particle-Mesh Ewald method. The equilibrated system was then subjected to a 500 ns production MD simulation using Parrinello-Rahman pressure coupling. Each simulation was performed three times. MD trajectories were analyzed using AmberTools 17 and visualized using PyMOL and VMD (54). The RMSD relative to the representative structure, the hydrogen-bond occupancies, and the interatomic distances described in this study were calculated for the first of the three trajectories using GROMACS tools (gmx rms, gmx hbond, and gmx distance). Hydrogen bonds between Mnt2p and α-Man-(1 → 3)-α-Man-OMe were defined by a donor–acceptor distance of ≤ 3.5 Å and a hydrogen–donor–acceptor angle of ≤ 30°, and the occupancy of each hydrogen bond was defined as the fraction of the frames (11,251 frames at 40-ps intervals) in the last 450 ns in which it was present.

## Data availability

The atomic coordinates and structure factors for Mnt2p have been deposited in the Protein Data Bank (http://www.rcsb.org) under accession code 45PL. All data supporting the findings of this study are available within the article (including its supplementary information files). Source data are available from the corresponding author on reasonable request.

## Supporting information

This article contains supporting information.

## Conflict of interest

The authors declare that they have no conflicts of interest with the contents of this article.
