# Supplementary material for: Structural and biochemical basis for the formation of α-(1 → 3)-linked mannose residue in O-glycans of Saccharomyces cerevisiae
Source: J Biol Chem. 2026 Oct 8;302(11):113626. doi: 10.1016/j.jbc.2026.113626 (PMC13756512; doi:10.1016/j.jbc.2026.113626)
Supplement: Supplementary file 1 [file mmc1.docx]

**Structural and biochemical basis for the formation of α-(1→3)-linked mannose residue in *O*-glycans of *Saccharomyces cerevisiae***

Daisuke Hira^1^*, Chihiro Kadooka^1^, Takuji Oka^1^*

^1^ Department of Biotechnology and Life Sciences, Faculty of Biotechnology and Life Sciences, Sojo University, 4-22-1 Ikeda, Kumamoto 860-0082, Japan

^*^Corresponding Authors:

**Supporting Information**

**Tables S1–S2**

**Figures S1–S6**

**Table S1.** Crystal parameters and data-processing statistics.

|  | ScMnt2 Mn/GDP-form |
| --- | --- |
| Data statistics |  |
| Beamline | SPring-8 BL44XU |
| Wavelength (Å) | 0.9000 |
| Space group | *P*2_1_2_1_2_1_ |
| Unit-cell parameters |  |
| *a*, *b*, *c* (Å) | 130.866 131.203 169.550 |
| α, β, γ (º) | 90, 90, 90 |
| Resolution (Å) | 38.87‒2.40 (2.54‒2.40) |
| No. of total reflections | 799028 (131106) |
| No. of unique reflections | 114706 (18231) |
| Completeness (%) | 99.8 (99.2) |
| Mean I/sigma(I) | 7.55 (1.15) |
| R_merge_ (%) | 13.0 (141.3) |
| R_meas_(%) | 14.1 (152.2) |
| CC_1/2_ | 0.992 (0.777) |
| Multiplicity | 7.0 (7.2) |
| Refinement statistics |  |
| *R*_work_ (%) | 20.6 |
| *R*_free_ (%) | 23.3 |
| No. of non-H atoms |  |
| No. of protein and ligand atoms | 17167 |
| No. of solvent atoms | 103 |
| R.m.s. deviations from ideal values |  |
| Bond length (Å) | 0.006 |
| Bond angle (º) | 1.33 |
| Average *B*-factor (Å^2^) |  |
| Protein | 79.6 |
| Solvent | 61.1 |
| Ramachandran plot, residues in |  |
| Favoured (%) | 96.69 |
| Allowed (%) | 3.31 |
| Outliers (%) | 0.00 |

Values in parentheses are for the highest resolution shell.

**Table S2.** Occupancy of hydrogen bonds between Mnt2p and the acceptor substrate model α-Man-(1→3)-α-Man-OMe during the last 450 ns of the MD simulation.

| **Mnt2p atom** | **Acceptor atom^a^** | **Occupancy (%)^b^** |
| --- | --- | --- |
| Asp-305 OD1 | O6 | 99.7 |
| Asp-305 OD2 | O4 | 98.7 |
| Arg-306 NH1 | O6 | 95.6 |
| Tyr-524 OH | O5 (ring oxygen) | 68.1 |
| Lys-399 NZ | O3 | 44.1 |
| Arg-306 NH1 | O2 | 42.1 |
| Lys-399 NZ | O4 | 37.0 |
| Asp-305 OD2 | O6 | 8.6 |
| Tyr-524 OH | O4 (Man-OMe) | 7.0 |

^a^ Oxygen atoms of the terminal (non-reducing) mannose residue unless otherwise indicated. ^b^ Fraction of the 11,251 frames (50–500 ns, 40-ps interval) in which the donor–acceptor distance was ≤ 3.5 Å and the hydrogen–donor–acceptor angle was ≤ 30°. Pairs with occupancy below 5% are not listed. Occupancy of hydrogen bonding of each residue to any acceptor atom: Asp-305, 100%; Arg-306, 96.7%; Tyr-524, 79.7%; Lys-399, 79.4%.

**Figure S1.** Structural comparison of Mnt2p with other GTs. **A.** Ribbon representation of the Mnt2p structure. The Mn^2+^ ion and GDP-Man molecule are shown as sphere and ball-and-stick models, respectively. The N-terminal and C-terminal domains of Mnt2p are color-coded green and magenta, respectively, and the N-termini and C-termini are labeled. **B.** Superimposed ribbon representations of the structures of Mnt2p (green and magenta) with WbbM bifunctional glycosyltransferase (PDB code 6U4B, cyan). **C.** Superimposed ribbon representations of the structures of Mnt2p (green and magenta) with glycogenin-1 (PDB code 1ZCT, cyan). **D.** Superimposed ribbon representations of the structures of Mnt2p (green and magenta) with the catalytic domain of UDP-Glucose: glycoprotein glucosyltransferase (UGGT) from *Chaetomium thermophilum* (PDB code 7ZLE, cyan). The α/β structure of the N-terminal domain of Mnt2p can be partially superimposed on the structures of other GTs, but a similar structure in the C-terminal domain of Mnt2p is not found in other GTs. Cα RMSD values from the DALI alignments: WbbM (6U4B, chain A), 4.4 Å over 204 aligned residues (the aligned residues lie predominantly within the C-terminal GT8 domain, residues 267–502); glycogenin-1 (1ZCT, chain A), 2.8 Å over 161 aligned residues; UGGT (7ZLE, chain A), 3.1 Å over 189 aligned residues.

**Figure S2.** Conformation analyses of the MD simulation of Mnt2p in complex with GDP-Man and α-Man-(1→3)-α-Man-OMe. **A.** Root mean square deviation (RMSD) analysis of the protein main chain (black), Mn^2+^ ion (magenta), GDP-Man (green) as donor substrate, and acceptor substrate model compound, α-Man-(1→3)-α-Man-OMe (cyan) during a 500-ns MD simulation. **B.** Root mean square fluctuation (RMSF) of the main chain atoms against the starting structure were calculated over the final 450 ns time. Some peaks in the plot indicate that the loop regions were a little flexible, suggesting relatively large fluctuations in the C-terminal domain. C. RMSD of the heavy atoms of α-Man-(1→3)-α-Man-OMe (cyan) and GDP-Man (green) relative to their conformations in the representative structure of the most populated cluster (Fig. 4B; t = 319 ns), calculated after superposition of each frame on the protein Cα atoms.

**Figure S3.** Time courses of the donor–acceptor distances between Mnt2p residues and the acceptor substrate model, α-Man-(1→3)-α-Man-OMe, during the last 450 ns of the MD simulation. The distances between the side-chain atoms of Asp-305 (OD1 or OD2), Arg-306 (NH1), Lys-399 (NZ), and Tyr-524 (OH) and the hydroxyl oxygen atoms (O2, O3, O4, and O6) or the ring oxygen atom (O5) of the terminal (non-reducing) mannose residue of α-Man-(1→3)-α-Man-OMe are shown; all oxygen atoms shown belong to the terminal mannose residue. The distances were mostly 3.5 Å or less, and the occupancy of each hydrogen bond is listed in Table S2.

**Figure S4.** High-resolution ESI-MS analysis of the acceptor substrate and the reaction products of Mnt2p. Negative-ion ESI mass spectra of the acceptor substrate α-Man-pNP (top; the two peaks eluting at ~8 and ~10 min in Fig. 1B were collected together), peak-A (middle), and peak-B (bottom) purified by HPLC. The compounds were detected as formate adduct ions, [M + HCOO]⁻. The observed m/z values of 346.0780, 508.1308, and 670.1836 agree with those calculated for the formate adducts of α-Man-pNP (C12H15NO8, monoisotopic mass 301.0798; [M + HCOO]⁻ calcd. 346.0780), α-Man-pNP with one additional mannose residue (C18H25NO13, 463.1326; calcd. 508.1308), and α-Man-pNP with two additional mannose residues (C24H35NO18, 625.1854; calcd. 670.1836), respectively, indicating that peak-A and peak-B are the products with one and two mannose residues added to α-Man-pNP.

**Figure S5.** Time courses of the distances between the 3-OH oxygen atom (O3) of the terminal mannose residue of α-Man-(1→3)-α-Man-OMe and the reactive groups of GDP-Man during the last 450 ns of the MD simulation. A. O3 to the C1 atom of the mannose moiety of GDP-Man. B. O3 to the bridging oxygen atom between the β-phosphate and the mannose C1 atom (the leaving-group oxygen). C. O3 to the non-bridging oxygen atom of the β-phosphate that is not coordinated to Mn^2+^. The other non-bridging oxygen atom of the β-phosphate is coordinated to Mn^2+^ and is not shown. The insets show the atom pairs whose distances were measured (double-headed arrows) in the representative structure of the most populated cluster (Fig. 4B); the Mn^2+^ ion is shown as a purple sphere. The mean ± SD values over the last 450 ns are 3.9 ± 0.4, 4.9 ± 0.4, and 4.6 ± 0.6 Å for A, B, and C, respectively.

**Figure S6.** Structural comparison of Mnt2p with AlphaFold models of the other GT71 mannosyltransferases of *S. cerevisiae*. The AlphaFold Database models (cyan) of the α-(1→3)-mannosyltransferases Mnn1p (A; UniProt P39106) and Mnt3p (B; P40549) and of the α-(1→2)-mannosyltransferases Mnn2p (C; P38069) and Mnn5p (D; P46982), from which the N-terminal transmembrane helix and its flanking regions had been removed, were superimposed on the crystal structure of Mnt2p (N-terminal domain, green; C-terminal domain, magenta; Mn^2+^ ion, sphere; GDP-Man, ball-and-stick model) using the super command of PyMOL. The models of Mnn1p and Mnt3p superimpose well on Mnt2p, including the C-terminal domain, whereas the models of Mnn2p and Mnn5p superimpose poorly on Mnt2p, including the region corresponding to the C-terminal domain.
